# The Development of a Screening Tool for Chinese Disordered Gamers: The Chinese Internet Gaming Disorder Checklist (C-IGDC)

**DOI:** 10.3390/ijerph17103412

**Published:** 2020-05-14

**Authors:** Juliet Honglei Chen, Meng Xuan Zhang, Chih-Hung Ko, Kwok Kit Tong, Shu M. Yu, Elvo Kuai Long Sou, Anise M. S. Wu

**Affiliations:** 1 Department of Psychology, Faculty of Social Sciences, University of Macau, Macao, China; JulietHChen@outlook.com (J.H.C.); yb77304@connect.um.edu.mo (M.X.Z.); kktong@um.edu.mo (K.K.T.); mogu.yus@gmail.com (S.M.Y.); 2 Center for Cognitive and Brain Sciences, University of Macau, Macao, China; 3 Department of Psychiatry, Kaohsiung Municipal Siaogang Hospital, Kaohsiung Medical University, Kaohsiung City 80708, Taiwan; chihhungko@gmail.com; 4 Student Affairs Office, University of Macau, Macao, China; elvosou@um.edu.mo

**Keywords:** addiction, Chinese, internet gaming disorder, psychometric properties, screening

## Abstract

Despite the increasing research attention being paid to gaming disorder globally, a screening tool developed specifically for the Chinese population is still lacking. This study aims to address this gap by constructing a screening tool to assess Internet gaming disorder (IGD) symptomology, defined by the fifth edition of the Diagnostic and Statistical Manual of Mental Disorders (DSM-5), among Chinese gamers. Based on expert interviews and consultations, a focus group of gamers, a background literature review, and the IGD criteria proposed by the DSM-5, we developed the Chinese Internet Gaming Disorder Checklist (C-IGDC). This study evaluated its dimensional structure, reliability, validity, and screening efficacy with 464 Chinese past-year gamers (53% female; mean age = 19.84). The two-level structure of the 27-item C-IGDC showed a satisfactory model fit, acceptable reliability, as well as good validity via expected associations with Internet addiction, gameplay frequency, and depressive symptoms. The optimal screening cutoff score (≥20) was proposed to detect probable IGD cases. The C-IGDC is the first DSM-5-based, multidimensional IGD screening tool designed specifically for Chinese gamers. Further evaluation of the C-IGDC in epidemiological studies and clinical settings is recommended.

## 1. Introduction

The recognition of gaming disorder as a mental disorder has generated increasing attention in clinical and research fields in recent years. The American Psychiatric Association [1] has proposed Internet gaming disorder (IGD) as a potential addictive disorder in the fifth edition of the Diagnostic and Statistical Manual of Mental Disorder (DSM-5), and more recently, the World Health Organization has included gaming disorder (predominantly online or offline) in the International Classification of Diseases 11th Revision (ICD-11) [2]. According to both the DSM-5 and the ICD-11, gaming disorder is a persistent (for at least 12 months, in most cases) and maladaptive pattern of gaming behavior characterized by an individual’s propensity to prioritize gaming over other activities, a loss of control over one’s gaming, and continuation of gaming despite negative consequences. The DSM-5 listed additional criteria for IGD, including preoccupation, withdrawal, tolerance, mood modification, deceiving others about one’s gaming behaviors, and problems regarding social relationships, jobs, or study. Despite the increasing research attention on IGD in recent years, assessment tools developed in the Chinese context are relatively uncommon. This study therefore aimed to develop a screening tool to facilitate the screening or early identification of probable IGD based on the DSM-5 formulation in a Chinese gaming population.

A common practice in screening for problematic gaming and IGD has been the use of instruments modified from scales for Internet addiction (IA; e.g., Young Internet Addiction Scale (YIAS) and Young Internet Addiction Test (YIAT)) [3,4], or other addictive behaviors (e.g., substance dependence, exercise addiction, and pathological gambling) [5,6,7]. These assessment tools share the limitation of using inconsistent screening criteria, which may result in an unreliable or even invalid estimation of IGD rate and misidentification of risk/protective correlates in a population [7,8]. Similar problems are evident in scales that have been developed or adapted for Chinese populations, such as the 29-item Online Gaming Addiction Scale for Adolescents which was modified from the YIAS [9]. This typical approach of adapting scales developed for other behaviors is likely to impact on the past-year assessment of problematic gaming and IGD in China. Similarly, the common reliance on all-purpose ‘Internet addiction’ tools (e.g., Young Diagnostic Questionnaire [4]) raises questions of validity for IGD-specific screening in this region [10], because these tools often include items that may be less relevant to problematic gaming.

To address the limitation of inconsistent screening criteria, a possible solution would be to adapt one of the popular Western assessment tools based on the DSM-5 proposed nine criteria of IGD into the Chinese context. However, there is no clearly optimal screening tool identified by the latest systematic review, given the varied strengths and weaknesses of the evidence across a total of 32 tools under review [10]. In studies that have adapted a Chinese version, there have usually been some drawbacks. Some adaptations, such as the Chinese adaptation of the Ten-Item Internet Gaming Disorder Test [11], for example, have an unknown factor structure in the Chinese context. Some other adaptations involved making structural modifications to the original scale in order to achieve satisfactory model fit, such as the Chinese version of the Internet Gaming Disorder Scale-9 Short Form [12] and the Chinese version of the Internet Gaming Disorder-20 Test [13]. These changes suggest that direct translations of items from foreign languages to Chinese may not be optimal, which underscores the need for a specific screening tool for Chinese disordered gamers. Scale development following procedures such as focus groups and expert panels can overcome many of the above limitations.

Given the aforementioned research gap, this study aimed to develop the Chinese Internet Gaming Disorder Checklist (C-IGDC), a screening tool that specifically designed to capture the problematic gaming characteristics of Chinese people. Corresponding to previous studies [7,13], we included IA, weekly gameplay frequency, and depressive symptoms as correlates. IA was treated as a potential indicator of convergent validity because IA has been regarded as an umbrella term that encompasses other specific problematic online behaviors (e.g., Internet gaming) [14]. Although a high level of gaming involvement may not be problematic for some players [15,16], we nevertheless expected to observe a positive association of IGD with weekly gameplay frequency as it has been consistently found before, and a similar association with depressive symptoms [7]. Furthermore, we also aimed to identify a screening cut-off score with the DSM-5 classification of IGD for early identifications of probable Chinese IGD cases and subsequent applications in a two-stage epidemiological evaluation for health researchers and practitioners.

## 2. Materials and Methods 

### 2.1. Item Construction of the C-IGDC

The initial item pool was constructed from three sources. We first recruited six Chinese IGD experts (e.g., clinical psychologists) through referrals for in-depth individual interviews to gather data regarding their experience of detecting, screening, and diagnosing disordered gaming cases. Meanwhile, six Chinese adults who self-reported ≥5 IGD symptoms listed in the DSM-5 were invited to attend a focus group, in which their gaming experience, especially with potentially disordered gaming symptoms, was explored. Thirdly, we conducted a comprehensive literature review on all the existing validated measures that assess IGD, such as Chinese Internet Addiction Scale [17]. Based on the information gathered from these three sources, it was concluded that a new IGD assessment tool should include a full coverage of the DSM-5 criteria. Specifically, we came up with a two-level, multidimensional IGD model conceptualization: one second-level parent-construct (i.e., C-IGDC) and nine first-level subconstructs (i.e., preoccupation, withdrawal, tolerance, unsuccessful to control, loss of interests, continued gaming despite psychosocial problems, deception, escape/relief, and problems). With this framework, we constructed 68 items as the initial sample pool.

In the following stage, four additional Chinese experts of addictive behaviors were invited to each make an independent assessment of the initial 68 items with a quantitative approach to content validity adapted from Lawshe [18], as well as participate in a subsequent joint expert group discussion on item refinement and further selection. After acquiring a consensus from all the experts, we composed a 34-item C-IGDC with satisfactorily high content validity endorsed by all the experts in identifying probable IGD cases among Chinese gamers. A subsequent pilot test, with a qualitative item analysis method adapted from Cohen et al. [19], was conducted among ten Chinese participants to understand the test performance from the perspective of test-takers. Based on the positive feedback provided by all the participants who considered the test as generally easy-to-understand and well-composed, we decided that the 34-item C-IGDC was ready for final testing among a broader range of the target population.

### 2.2. Respondents and Procedures

In the final stage of scale development, we assessed the structure, validity, and reliability of the 34-item C-IGDC among 464 Chinese undergraduate past-year gamers (53% female; age = 17 to 25 years, *M* = 19.84, *SD* = 1.50). All the participants were solicited through the undergraduate subject pool system at a public university in Macao, China in 2017/18 with the recruitment criteria of Chinese ethnicity, both sexes, and having Internet gaming experience in the past year. The participation was totally voluntary without any monetary compensation. Eligible participants who registered to this study via the online subject pool system were briefed on the study purpose and their rights by a trained research assistant in a classroom during the time slot they selected. The self-report questionnaire was only distributed to and completed by consented participants (informed consent was also obtained from their legal guardian for those under age of 18 years). This study was conducted with approval from the research ethics committee of the affiliated institute of the corresponding author (ethical approval no.: MYRG2016-00162-FSS) and in accordance with the latest version of the Declaration of Helsinki.

### 2.3. Measures

Participants were asked to state their sex, age, gaming behaviors (i.e., weekly gameplay frequency, monetary expenditure on games, and gaming device preference), and to respond to the following scales, in which a higher score indicated a higher level of the corresponding construct measured. Summation scores were calculated for each scale.

#### 2.3.1. C-IGDC

Participants responded to 34 items regarding the frequency of their past-year Internet gaming behaviors on a 3-point Likert response scale (0 = never, 1= sometimes, and 2 = often). A sample question is, “How often did you feel anxious and/or irritated while not being able to play Internet games (in the past 12 months)?”. Further psychometric evaluation and refinement of the C-IGDC are detailed in the following sections.

#### 2.3.2. Diagnostic Criteria of IGD in the DSM-5

Nine items from the DSM-5′s IGD criteria were used to assess IGD symptoms participants may have experienced over the past 12 months. They responded to the items on a dichotomous scale (1 = yes, 0 = no), with a sample item being, “Have you made unsuccessful attempts to control the participation in Internet games (in the past 12 months)?”. The scale showed satisfactory reliability in this study (KR-20 = 0.74). As guided by the DSM-5 [1] and previous studies that evaluated/used this diagnostic criteria in Chinese samples for diagnostic purposes [20,21], a cutoff of ≥5 was adopted to detect probable IGD cases as a reference to assess the screening efficacy of the C-IGDC and was also used to identify the cutoff score of the C-IGDC.

#### 2.3.3. IA Symptoms

YIAT [4] was used to measure addiction symptoms regarding general online behaviors in the past 12 months. Participants responded to the 20 items (e.g., “How often do you find that you stay on-line longer than you intended?”) on a five-point Likert questionnaire (1= rarely to 5 = always), with an overall score ranging from 20 to 100. The Chinese version of this test showed high reliability in previous studies with young adult samples [22]. The reliability of YIAT in this study was 0.90. A cutoff of ≥50, consistent with previous Asian studies on college students’ gaming [23,24], was selected to identify probable IA cases in the present sample.

#### 2.3.4. Depressive Symptoms

Depressive symptoms were assessed by the 10-item short version of the Center of Epidemiological Studies Depression Scale [25], in which participants were asked to respond to the items based on their experience during the past week on a four-point Likert response scale (0 = never or seldom to 3 = always). A sample item is, “I felt that people disliked me”. The scale showed good reliability in the current study (*α* = 0.88).

### 2.4. Statistical Analyses

When testing the structure and dimensionality of the C-IGDC with confirmatory factor analysis (CFA), we treated the C-IGDC items as ordinal variables and chose robust weighted least squares estimation mean and variance adjusted method, which is preferred estimation method for ordinal variables [26]. With Mplus 7.4 [27], we specified the CFA as a second-order factor analysis model. The C-IGDC, the second-order construct, consists of nine first-order subconstructs that each correspond to one of the nine DSM-5-proposed IGD diagnostic criteria and is measured with three to five items. The goodness-of-fit of the model, assessed by CFI ≥ 0.95, TLI ≥ 0.95, RMSEA ≤ 0.08, and SRMR ≤ 0.08, was satisfactory [28,29]. Once the model structure of the 34-item C-IGDC was tested, we further extracted three items with the highest standardized factor loadings from each first-order subconstruct to form a 27-item alternative second-order factor model (the 27-item C-IGDC) to ensure an equal weight of each subconstruct in relation to the second-order construct. After weighing the performance of the two versions of the C-IGDC, one superior version was selected to conduct subsequent reliability and validity tests with SPSS 25.0 [30]. Reliability was assessed with Cronbach’s alpha, whereas validity was tested by correlations to IA symptoms, weekly gameplay frequency, and depressive symptoms.

To estimate an optimal cutoff score for the C-IGDC for screening potential IGD cases, we first conducted a receiver operating characteristics (ROC) analysis on the C-IGDC to obtain the DSM-5 IGD-referenced area under the curve (AUC) as a general indicator of the screening efficacy of the C-IGDC for screening potential IGD cases. Subsequently, to compare with the DSM-5 IGD classification (self-reported score ≥ 5), we computed additional screening efficacy indices with DAS_STAT [31], including sensitivity, specificity, positive predictive rate (PPR), negative predictive rate (NPR), Cohen’s κ, Youden’s index, and diagnostic odds ratio (DOR). Cohen’s κ indicates whether the agreement between measurements is poor (≤0.40), fair (0.41–0.60), good (0.60–0.74), or excellent (≥0.75) [32]. Youden’s index takes sensitivity and specificity into account in equal parts, with a higher score reflecting a higher combined sensitivity and specificity rate [33]. Higher values of DOR suggest better discriminatory test performance, regardless of the prevalence of the target disorder [34]. The optimal cutoff score was screened from a starting point of both sensitivity and specificity rates higher than 75% [35]. Within this possible range, all the efficacy indices were taken into account to balance a high sensitivity (i.e., to identify as many positive cases as possible), a relatively high specificity (i.e., to keep true negative rate at an acceptable level), and a high Cohen’s κ, (i.e., to maintain the highest level of measurement agreement at a possible range), in order to meet the goal of screening for probable IGD cases. We also tested the discriminant validity of the proposed screening cutoff point of the C-IGDC by dividing the overall sample into a probable IGD group and a non-IGD group for further between-group comparisons.

## 3. Results

### 3.1. Descriptive Statistics 

In our gamer sample (*N* = 464), 59.5% played more than three days a week and 23.3% played less than one day a week. About 2/3 of the respondents (67.7%) did not spent money on Internet gaming, whereas 11.4% spent more than 100MOP (12.4USD) per month. The majority (59.3%) of the respondents played mostly on smartphones, followed by computers (31.5%), tablets (6.7%), and gaming consoles (2.6%).

### 3.2. Structure and Dimensionality of the C-IGDC

We first tested the one-factor unconstrained model of the 34 C-IGDC items and found its model fit to be unsatisfactory, χ^2^(527) = 2089.81, *p* < 0.001, CFI = 0.87, TLI = 0.86, RMSEA = 0.080 (90% CI [0.076, 0.084]), SRMR = 0.091. The proposed second-order factor structure of the 34-item model showed a more satisfactory model fit, χ^2^(518) = 1042.65, *p* < 0.001, CFI = 0.96, TFL = 0.95, RMSEA = 0.047 (90% CI [0.043, 0.051]), SRMR = 0.068. We then performed another CFA on the 27-item version, which was composed of nine sets of three items that carried the highest standardized factor loadings within each first-order subconstruct of the 34-item version (see Table 1). With the same second-order factor structure, the 27-item version displayed a comparatively satisfactory model fit as the 34-item one, χ^2^(315) = 657.66, *p* < 0.001, CFI = 0.97, TLI = 0.96, RMSEA = 0.048 (90% CI [0.043, 0.054]), SRMR = 0.063, which suggested that the two versions of the C-IGDC were of similarly good structure validity. The standardized factor loadings of each item are shown in Table 1.

For the sake of parsimony and ensuring an equal weight of each subconstruct in relation to the higher order construct, the 27-item version (hereafter referred to as the C-IGDC; see Appendix A for the Chinese version) was selected for further reliability and validity examinations. It should also be noted that the one-factor unconstrained model of this 27-item version had an unsatisfactory model fit, χ^2^(324) = 1568.83, *p* < 0.001, CFI = 0.87, TLI = 0.86, RMSEA = 0.091 (90% CI [0.087, 0.096]), SRMR = 0.091.

### 3.3. Reliability, Between-Factor Associations, and Validity

As shown in Table 2, the C-IGDC demonstrated high reliability (*α* = 0.92) in the current sample, whereas its nine subconstructs also displayed acceptable reliability (*α* = 0.65 to 0.79). All of the nine subconstructs manifested a strong positive association with the C-IGDC (*r* = 0.62 to 0.80, *p* < 0.001) and a moderate-to-strong positive between-factor association with each other (*r* = 0.34 to 0.62, *p* < 0.001). In terms of validity, the C-IGDC showed a moderate positive correlation with IA symptoms (*r* = 0.45, *p* < 0.001) and weekly gameplay frequency (*r* = 0.40, *p* < 0.001), and a mild positive correlation with depressive symptoms (*r* = 0.28, *p* < 0.001). Furthermore, all nine subconstructs of the C-IGDC showed positive, significant associations with IA (*r* = 0.22 to 0.39, *p* < 0.001), gameplay frequency (*r* = 0.14 to 0.41, *p* < 0.001), and depressive symptoms (*r* = 0.12 to 0.29, *p* < 0.001).

### 3.4. Screening Efficacy and Setting the Optimal Screening Cutoff Point of the C-IGDC

The screening efficacy was operationalized as AUC from the ROC analysis. We adopted the DSM-5 IGD cutoff point of ≥5 as reference [1] to conduct ROC analysis on the C-IGDC and found a good efficacy (AUC = 0.91). The process of setting the optimal cutoff point of the C-IGDC was carried out in two steps. As guided by Lowe [35], we first listed all the potential cutoff points with both sensitivity and specificity rates greater than 75% (see Table 3), which generated five possible candidates from 17 to 21. Then, we narrowed the list down to two possible cutoffs points (i.e., ≥20 and ≥21), with the highest Cohen’s κ of 0.50 among the five, for further comparison. As the C-IGDC is designed as a screening tool, we finally chose the point of ≥20 as the optimal screening cutoff point of the C-IGDC for having a higher level of sensitivity, Youden’s index, and DOR, compared to the other possible cutoff point (i.e., ≥21). Using this cut-off, the estimated probable IGD proportion was 23.3% in our gamer sample. It is lower than the percentage of probable IA cases (i.e., 33.2%; YIAT ≥ 50) and higher than the IGD ratio estimated with the nine DSM-5 diagnostic criteria (i.e., 12.9%; DSM-5 ≥ 5).

After dividing the overall sample into the potential IGD group and the non-IGD group according to the C-IGDC and the cutoff of ≥20, we identified significant between-group differences on IA symptoms (*M*_IGD_ = 54.27, *M*_non-IGD_ = 42.36, *t*(462) = 9.33, *p* < 0.001), depressive symptoms (*M*_IGD_ = 12.04, *M*_non-IGD_ = 8.80, *t*(462) = 5.16, *p* < 0.001), and DSM-5 IGD symptoms (*M*_IGD_ = 4.22, *M*_non-IGD_ = 1.35, *t*(462) = 15.91, *p* < 0.001). In contrast, similar between-group differences were found in the current sample when applying the DSM-5 IGD cutoff of ≥5 to differentiate potential IGD and non-IGD groups (IA symptoms: *M*_IGD_ = 57.02, *M*_non-IGD_ = 43.37, *t*(462) = 8.36, *p* < 0.001; depressive symptoms: *M*_IGD_ = 12.30, *M*_non-IGD_ = 9.15, *t*(462) = 3.95, *p* < 0.001; C-IGDC IGD symptoms: *M*_IGD_ = 25.30, *M*_non-IGD_ = 11.30, *t*(462) = 13.81, *p* < 0.001).

Moreover, we compared the game-playing habits between the probable IGD and non-IGD groups. The C-IGDC-identified probable IGD gamers were found to play more frequently (*U* = 12,059.0, *p* < 0.001) and spend more money (*U* = 13,656.0, *p* < 0.001) on games, but they displayed no significant preference over gaming devices (χ^2^(3) = 0.70, *p* = 0.87) when compared to non-IGD gamers; the probable IGD gamers identified by the DSM-5 manifested the exact same tendencies of higher weekly gameplay frequency (*U* = 7677, *p* < 0.001) and monetary expenditure (*U* = 9767, *p* = 0.003), with no device preference (χ^2^(3) = 1.82, *p* = 0.61). In sum, probable IGD gamers, regardless of whether they were screened by the C-IGDC or the DSM-5 IGD criteria, reported similar patterns of significantly higher IA severity, more depressive symptomology, higher weekly gameplay frequency, more monetary expenditure on games, and no gaming device preference when compared to their non-IGD counterparts.

## 4. Discussion 

In this study, we reported the development process and psychometric properties of the 27-item C-IGDC, which is the first multidimensional, DSM-5-based IGD screening tool developed in a Chinese adult population. The two-level IGD model structure showed a satisfactory model fit, acceptable reliability, and expected validity in our Chinese gamer sample. Specifically, the dimensional structure of the C-IGDC showed generally high factor loadings across all the subconstructs, with the exception of deception (F7) and escape and relief (F8), which contributed relatively less to the parent C-IGDC construct; these results have been also observed in other DSM-5-based IGD scales [36]. One reason for such a pattern may be that escape or relief appears to be relatively common among non-disordered gamers [36,37], whereas deception is more likely to occur in severe IGD cases [38] or may not be applicable in cases of gaming in social isolation or under constant social surveillance by a parent or partner. For reliability, the overall scale of the C-IGDC displayed a high internal consistency (*α* = 0.92), whereas its nine subscales presented relatively lower but still acceptable reliability (*α* = 0.65 to 0.79), presumably due to a small number of items (i.e., three) in each. Moreover, the convergent validity of the C-IGDC was identified by its moderate association with IA. In addition, the observed moderate association with weekly gameplay frequency and mild association with depressive symptoms were consistent with previous studies [13,39,40].

The C-IGDC also displayed a good overall screening efficacy in detecting DSM-5-proposed IGD cases (AUC = 0.91). Because the use of the C-IGDC for screening probable IGD was the primary purpose of this study, it was desirable to identify a cutoff point with the balanced sensitivity, specificity, and agreement between measures. By setting ≥20 as the screening cutoff point, we found the efficacy of the C-IGDC (81.7% sensitivity and 85.4% specificity) was optimized in screening probable IGD cases among Chinese people. This efficacy, with respect to sensitivity and specificity, is comparable to what was proposed by Ko et al. [41] (83.9% sensitivity and 76.7% specificity) for the Chen Internet Addiction Scale (CIAS), which is the closest analog to the C-IGDC, as both are screening tools of Internet-related addiction that were indigenously developed from Chinese samples. When applying this cutoff point, the C-IGDC-identified probable IGD gamers showed significantly more symptoms of IA, depression, and DSM-5 IGD, which resembled how the DSM-5-identified IGD gamers behaved and hence further confirmed the C-IGDC as a valid screening tool for DSM-5 proposed IGD criteria. Although the C-IGDC identified more probable IGD cases than those classified by the nine-item DSM-5 criteria in the present Chinese university gamer sample (23.3% versus 12.9%), it serves a primary screening purpose, given the generally high but varying rate of IGD among Chinese university students (e.g., 3.7–17.0% [42,43]). This high estimated percentage of probable IGD in our sample should not be treated as a ‘prevalence’ estimate of Chinese disordered gamers in the community, given the convenience sampling and the selective inclusion of young gamers, as well as the relatively higher rate of endorsement of DSM-5 IGD items among young Asian gamers. The proportion of probable IGD cases detected by the C-IGDC also fell within the expected range of its umbrella disorder, IA (YIAT ≥ 50; 33.2%) in the present sample. Similar between-group differences on gaming habits (i.e., gameplay frequency, monetary expenditure on games, and gaming device preference) were also observed for both C-IGDC-identified and DSM-5-identified probable IGD gamers when compared with their non-IGD counterparts, in line with previous studies [13,39].

The C-IGDC aims to identify potential disordered gamers in the Chinese population, but a symptomatic evaluation on IGD reveals only a partial, symptom-based picture of the clinical condition of disordered gamers [44]. Subsequent studies may consider integrating the C-IGDC with alternative approaches, such as those focused on key psychological processes (e.g., motivation [45]), as well as personality traits and gaming-related harms [46], in order to further uncover potential subgrouping patterns of disordered gamers, and thus inform prognosis and better treatment planning. Despite the ongoing debate on whether daily life behaviors, such as gaming and social networking, are over-pathologized as mental disorders [44,47,48], there is accumulating evidence that supports the consideration of gaming disorder as an addiction disorder [49,50]. However, a stronger theoretical and methodological basis for gaming disorder or other behavioral addictions across populations, such as building up a more standardized functioning impairment assessment system and providing more longitudinal evidence of the stability of these dysfunctional behaviors, is warranted [44]. We hope the development of the C-IGDC can facilitate further empirical investigations into Chinese gaming behaviors.

The present study also had some limitations and unsolved issues. Firstly, the convenience sampling limited the extent of generalizability of our findings on the psychometric soundness of the C-IGDC to broader populations. The observed number of probable IGD cases in this study, albeit with a convenience sample selected for gaming, was quite high. The DSM-5 approach appears to be more sensitive than the ICD-11 with its inclusion of symptoms such as tolerance and withdrawal, which likely contributes to higher endorsement. We call for further validation of the C-IGDC to be conducted with a more general gamer sample across different demographic and player groups, especially with respect to the efficacy of the currently proposed screening cutoff point. The inclusion of items that screen for severe harms due to gaming might be a useful cross-validation approach. Secondly, probable self-report bias is inevitable when using survey methods, and hence future studies should consider collecting additional behavioral data for comparison. Thirdly, we also recommend applying the C-IGDC to assist in a two-stage epidemiological evaluation of IGD for first-step screening and comparing the C-IGDC score with clinical diagnosis results to compute a diagnostic cutoff point in further studies.

## 5. Conclusions

The C-IGDC is the first DSM-5-based, multidimensional IGD screening tool developed specifically for the Chinese population. The present findings supported the psychometric soundness of the C-IGDC in screening probable IGD cases among Chinese young adults. Although additional studies are needed to confirm its screening power in other populations (e.g., primary/secondary school students and older adults), the C-IGDC presents promising potential in assisting health researchers and practitioners’ early identification of probable IGD cases. It also provides a useful alternative to the all-purpose Internet addiction tools (e.g., YIAT) that are often used in China and other Asian contexts. We also recommend that future studies apply the C-IGDC in a two-stage epidemiological evaluation of IGD among Chinese gamers and evaluate this tool for its potential clinical utility.

## Figures and Tables

**Table 1:** Confirmatory factor analysis results of two versions of the Chinese Internet Gaming Disorder Checklist (C-IGDC) (*N* = 464).

Sub-Construct/Item	Standardized Factor Loadings	
	34-Item	27-Item
**F1: Preoccupation**	**0.75** (0.57)	**0.78** (0.60)
F1-1. Preoccupation with Internet games	0.88	0.87
F1-2. Always anticipate playing Internet games again while not playing	0.83	0.81
F1-3. Involuntarily imagine things happened in the Internet games while not playing	0.82	0.79
F1-4. Involuntarily think about things related to Internet games	0.81	--
**F2: Withdrawal**	**0.90** (0.81)	**0.90** (0.80)
F2-1: Feel anxious and/or irritated while not being able to play Internet games	0.75	0.76
F2-2: Feel upset and/or distracted while not being able to play Internet games for any reason	0.80	0.81
F2-3: Feel angry when others interrupt your Internet gaming	0.62	--
F2-4: Feel like losing everything while not being able to play Internet games	0.90	0.92
**F3: Tolerance**	**0.91** (0.82)	**0.92** (0.85)
F3-1: Feel like Internet gaming is becoming more and more important to you	0.83	0.84
F3-2: Need to spend more and more time on Internet gaming to feel content	0.86	0.86
F3-3: Need continued collecting prizes, breaking records, and/or passing more levels to gain desired thrill and/or content	0.65	0.64
**F4: Unsuccessful to control**	**0.93** (0.87)	**0.97** (0.93)
F4-1: Have attempted at cutting down Internet gaming, but found it difficult	0.78	0.76
F4-2: Feel like you cannot stop Internet gaming	0.73	0.73
F4-3: Have planned Internet gaming for only a little while, but ended up with gaming for an extended longer period	0.68	--
F4-4: Have attempted to cut down or stop Internet gaming, but failed and started playing again	0.73	0.71
**F5: Loss of interests**	**0.86** (0.73)	**0.81** (0.65)
F5-1: Cannot enjoy other activities as much as before because of Internet gaming	0.83	0.87
F5-2: Decrease the amount of other recreational activities because of Internet gaming	0.83	0.86
F5-3: Put Internet gaming before other things	0.73	--
F5-4: Decrease the offline contacts with others because of Internet gaming	0.76	0.79
**F6: Continued gaming despite psychosocial problems**	**0.84** (0.70)	**0.83** (0.68)
F6-1: Continued gaming after you understand that Internet gaming has already negatively affected you	0.76	0.76
F6-2: Continued gaming under sleep deficiency	0.62	--
F6-3: Continued gaming despite receiving objections from your family	0.79	0.78
F6-4: Continued gaming for pursuing levels, breaking records, and etc. when you should take a break	0.74	0.70
**F7: Deception**	**0.71** (0.51)	**0.71** (0.51)
F7-1: Have deceived others about your enthusiasm for Internet gaming	0.80	0.81
F7-2: Have deceived others about your excessive amount of Internet gaming	0.95	0.95
F7-3: Have deliberately hidden things related to your Internet gaming from others	0.79	0.79
**F8: Escape/Relief**	**0.67** (0.44)	**0.68** (0.46)
F8-1: Use of Internet gaming as an escape from the reality	0.81	0.81
F8-2: Use of Internet gaming to relieve a bad mood	0.93	0.95
F8-3: Use of Internet gaming to forget your worries	0.89	0.90
**F9: Problems**	**0.84** (0.70)	**0.82** (0.68)
F9-1: Impaired significant relationship because of Internet gaming	0.73	--
F9-2: Disputes or conflicts with your family members or friends because of Internet gaming	0.76	0.76
F9-3: Impaired performance or efficiency at work/study, or even problems, because of Internet gaming	0.77	0.78
F9-4: Impaired significant aspects in your life because of Internet gaming	0.90	0.93
F9-5: Skip school or work because of Internet gaming	0.71	--

Note: The second-order standardized factor loadings are bolded. The R-squared value of each latent variable are in parentheses.

**Table 2:** Reliability and bivariate correlations of the C-IGDC and its nine sub-constructs (*N* = 464).

Construct/Sub-Construct	C-IGDC	F1	F2	F3	F4	F5	F6	F7	F8	F9
C-IGDC	(0.92)	--	--	--	--	--	--	--	--	--
F1: Preoccupation	0.70 **	(0.75)	--	--	--	--	--	--	--	--
F2: Withdrawal	0.72 **	0.47 **	(0.68)	--	--	--	--	--	--	--
F3: Tolerance	0.76 **	0.54 **	0.62 **	(0.65)	--	--	--	--	--	--
F4: Unsuccessful to control	0.80 **	0.50 **	0.55 **	0.58 **	(0.65)	--	--	--	--	--
F5: Loss of interests	0.72 **	0.37 **	0.55 **	0.50 **	0.53 **	(0.76)	--	--	--	--
F6: Continued gaming	0.73 **	0.50 **	0.41 **	0.50 **	0.56 **	0.44 **	(0.70)	--	--	--
F7: Deception	0.62 **	0.35 **	0.38 **	0.33 **	0.41 **	0.43 **	0.34 **	(0.76)	--	--
F8: Escape/Relief	0.69 **	0.39 **	0.37 **	0.49 **	0.51 **	0.36 **	0.43 **	0.38 **	(0.79)	--
F9: Problems	0.74 **	0.44 **	0.47 **	0.42 **	0.56 **	0.53 **	0.50 **	0.48 **	0.44 **	(0.72)
DSM-5 IGD symptoms	0.72 **	0.51 **	0.54 **	0.59 **	0.59 **	0.52 **	0.50 **	0.48 **	0.47 **	0.52 **
Internet addiction symptoms	0.45 **	0.22 **	0.37 **	0.34 **	0.37 **	0.38 **	0.28 **	0.28 **	0.30 **	0.39 **
Weekly gameplay frequency	0.40 **	0.41 **	0.20 **	0.32 **	0.35 **	0.17 **	0.42 **	0.14 **	0.25 **	0.31 **
Depressive symptoms	0.28 **	0.12 *	0.21 **	0.23 **	0.19 **	0.24 **	0.15 **	0.16 **	0.29 **	0.21 **

Note: Cronbach’s *α* is shown in diagonal parentheses. * p < 0.05, ** p < 0.01.

**Table 3:** Cutoff point of the C-IGDC based on the Diagnostic and Statistical Manual of Mental Disorders (DSM-5)-proposed diagnostic criteria of Internet gaming disorders (IGD) (*N* = 464).

Cutoff Point (≥)	Sensitivity (%)	Specificity (%)	PPR (%)	NPR (%)	Cohen’s κ	Youden’s Index	DOR
16	91.7	73.0	33.5	98.3	0.37	0.65	30.91
17	90.0	78.5	38.3	98.1	0.43	0.69	32.15
18	90.0	80.4	40.6	98.2	0.46	0.70	38.33
19	86.7	82.7	42.6	97.7	0.48	0.69	31.25
**20**	**81.7**	**85.4**	**45.4**	**96.9**	**0.50**	**0.67**	**26.62**
21	76.7	87.1	46.9	96.2	0.50	0.64	22.07
22	68.3	90.3	51.3	95.1	0.51	0.59	20.23

Note: Positive Predictive Rate (PPR); Negative Predictive Rate (NPR); Diagnostic Odds Ratio (DOR). The line in bold indicates the optimal cutoff score.

## Appendix A

### ***Chinese Internet Gaming Disorder Checklist (C-IGDC)*** ***華人網絡遊戲成癮篩查量表***

In the past 12 months, how often have you experienced the following situations? (0 = *never*, 1 = *sometimes*, 2 = *often*.) 請問在過去12個月，以下情況有多經常發生在你身上? (0 = 從不，1 = 有時， 2 = 常常)

F1: Preoccupation 因子1. 沈湎遊戲

(1) 你的腦海裡會充滿著關於網絡遊戲的事情嗎？
(2) 當你不玩網絡遊戲的時候，你會總惦記著下次再玩嗎？
(3) 當你不玩網絡遊戲的時候，你會不由自主地想象網絡遊戲中發生的事嗎？

F2: Withdrawal 因子2. 遊戲戒斷

(1) 你在不能玩網絡遊戲的時候，會感覺急燥易怒嗎？
(2) 不論因為任何原因不能玩網絡遊戲的時候，你會覺得心煩意亂嗎？
(3) 當你無法玩網絡遊戲的時候，你會感到像失去了所有或全部嗎？

F3: Tolerance 因子3. 遊戲耐受

(1) 你會感到玩網絡遊戲對你越來越重要嗎？
(2) 你會需要用越來越多的時間玩網絡遊戲才能滿足嗎？
(3) 你會需要不斷收集、打破記錄，或過關來獲得嚮往的興奮或滿足感嗎？

F4: Unsuccessful to control 因子4. 控制失敗

(1) 你試過想少玩點網絡遊戲，但感到很難做到嗎？
(2) 你會覺得你沒法不玩網絡遊戲嗎？
(3) 你試過減少甚至停止玩網絡遊戲，但嘗試失敗後又開始玩嗎？

F5: Loss of interests 因子5. 興趣減少

(1) 網絡遊戲會使你不再像以前一樣享受其他活動嗎？
(2) 你會因為網絡遊戲而減少參與其他消遣活動嗎？
(3) 你會因為玩網絡遊戲而減少了和別人的線下互動嗎？

F6: Continued playing despite psychosocial problems 因子6. 明知故玩

(1) 你會在明白玩網絡遊戲已經對你造成負面影響之後，繼續玩網絡遊戲嗎？
(2) 即使家人反對，但你還是會繼續玩網絡遊戲嗎？
(3) 你試過到了應該休息的時間, 但為了想過關或破紀錄等原因而繼續玩網絡遊戲嗎？

F7: Deception 因子7. 隱瞞欺騙

(1) 你試過對其他人欺瞞過你對網絡遊戲的熱衷程度嗎？
(2) 你試過因為玩網絡遊戲太多而對其他人說謊嗎？
(3) 你試過刻意對其他人隱瞞你玩網絡遊戲的一些事嗎？

F8: Escape/Relief 因子8. 情緒解脫

(1) 你會用玩網絡遊戲來逃避現實生活嗎？
(2) 你會用玩網絡遊戲來處理你的壞情緒嗎？
(3) 你會藉玩網絡遊戲來忘掉煩憂嗎？

F9: Problems 因子9. 問題行為

(1) 你試過因為玩網絡遊戲而與家人或朋友發生爭執或衝突嗎？
(2) 玩網絡遊戲會令你在工作(或學業)上的表現或效率變差，甚至出現問題嗎？
(3) 玩網絡遊戲會對你生活裡重要的事情產生負面影響嗎？
